# PTEN overexpression and nuclear β-catenin stabilization promote morular differentiation through induction of epithelial–mesenchymal transition and cancer stem cell-like properties in endometrial carcinoma

**DOI:** 10.1186/s12964-022-00999-w

**Published:** 2022-11-21

**Authors:** Ako Yokoi, Marina Minami, Miki Hashimura, Yasuko Oguri, Toshihide Matsumoto, Yoshinori Hasegawa, Mayu Nakagawa, Yu Ishibashi, Takashi Ito, Kensuke Ohhigata, Youhei Harada, Naomi Fukagawa, Makoto Saegusa

**Affiliations:** 1 Department of Pathology, Kitasato University School of Medicine, 1-15-1 Kitasato, Minami-Ku, Sagamihara, Kanagawa 252-0374 Japan; 2 Department of Pathology, Kitasato University School of Allied Health Science, 1-15-1 Kitasato, Minami-Ku, Sagamihara, Kanagawa 252-0374 Japan; 3 Laboratory of Clinical Omics Research, Department of Applied Genomics, Kazusa DNA Research Institute, 2-6-7 Kazusakamatari, Kisarazu, Chiba 292-0818 Japan

**Keywords:** PTEN, β-catenin, EBP50, Cancer stem cell, Endometrial carcinoma, Morule

## Abstract

**Background:**

Although a lack of functional PTEN contributes to tumorigenesis in a wide spectrum of human malignancies, little is known about the functional role of its overexpression in the tumors. The current study focused on PTEN overexpression in endometrial carcinoma (Em Ca).

**Methods:**

The functional impact of PTEN overexpression was assessed by Em Ca cell lines. Immunohistochemical analyses were also conducted using 38 Em Ca with morular lesions.

**Results:**

Em Ca cell lines stably overexpressing PTEN (H6-PTEN) exhibited epithelial–mesenchymal transition (EMT)-like features, probably through β-catenin/Slug-meditated suppression of E-cadherin. PTEN overexpression also inhibited cell proliferation, accelerated cellular senescence, increased apoptotic features, and enhanced migration capability. Moreover, H6-PTEN cells exhibited cancer stem cell (CSC)-like properties, along with high expression of aldehyde dehydrogenase 1 and CD44s, a large ALDH 1^high^ population, enriched spheroid formation, and β-catenin-mediated upregulation of cyclin D2, which is required for persistent CSC growth. In clinical samples, immunoreactivities for PTEN, as well as CSC-related molecules, were significantly higher in morular lesions as compared to the surrounding carcinomas. PTEN score was positively correlated with expression of nuclear β-catenin, cytoplasmic CD133, and CD44v6, and negatively with cell proliferation. Finally, estrogen receptor-α (ERα)-dependent expression of Ezrin-radixin-moesin-binding phophoprotein-50 (EBP50), a multifunctional scaffolding protein, acts as a negative regulator of morular formation by Em Ca cells through interacting with PTEN and β-catenin.

**Conclusion:**

In the abscess of ERα/EBP50 expression, PTEN overexpression and nuclear β-catenin stabilization promote the establishment and maintenance of morular phenotype associated with EMT/CSC-like features in Em Ca cells.

**Video Abstract**

**Supplementary Information:**

The online version contains supplementary material available at 10.1186/s12964-022-00999-w.

## Background

*PTEN (phosphatase and tensin homolog deleted on chromosome ten*) was ordinally identified as a tumor suppressor gene [1, 2]. Exerting its best understood biochemical function, PTEN acts as both an inositol phospholipid phosphatase and a dual protein phosphatase capable of dephosphorylating phospho-threonine, -serine, and -tyrosine, thereby antagonizing the phosphoinositide 3 kinase (PI3K)-AKT pathway [3, 4]. One major effector of PI3K-AKT signaling is mammalian target of rapamycin (mTOR), which stimulates protein synthesis, initiates entry into G1 phase of the cell cycle, and interacts with proteins that regulate apoptosis [4, 5].

Loss of PTEN function occurs in a wide spectrum of human malignancies through a variety of mechanisms, including mutations, deletions, promoter hypermethylation, loss of heterozygosity, aberrant microRNA expression, and increased protein instability [6]. For example, somatic *PTEN* mutations are frequently observed in 34–55% of endometrial carcinoma (Em Ca), particularly in endometrioid-type malignancies [7, 8]. Although it has been widely accepted that a lack of functional PTEN contributes to tumorigenesis by preventing apoptosis and increasing proliferative activity [9], little is known about the functional role of its overexpression in Em Ca cells.

Focal squamous differentiation into a morular phenotype, which is defined by incomplete or immature squamous differentiation, is a common event in endometrioid-type Em Ca [10]. We previously demonstrated that identical β-catenin mutations were detected in both morular and glandular lesions of Em Ca, and that nuclear β-catenin accumulation is a critical factor in triggering trans-differentiation towards the morular phenotype [11, 12].

Ezrin-radixin-moesin-binding phophoprotein-50 (EBP50) is a multifunctional scaffolding protein that exerts different functions in carcinomas through its interactions with oncogenic or tumor-suppressive proteins, including β-catenin and PTEN [13]. Given that Wnt/β-catenin and phosphatidylinositol-3 kinase (PI3K)/PTEN signaling pathways include GSK-3β as a common point of intersection [14], we hypothesized that crosstalk between PTEN, β-catenin, and EBP50 may participate in Em Ca cell morular formation. To test this, we investigated the expression of PTEN, β-catenin, and EBP50, with reference to epithelial–mesenchymal transition (EMT)- and cancer stem cell (CSC)-like features, using Em Ca cell lines and clinical samples.

## Methods

### Plasmids and cell lines

The human PTEN cDNA was kindly provided from Dr Suzy Baker (St. Jude Children’s Hospital) and was subcloned into the pcDNA3.1 vector (Invitrogen, Carlsbad, CA, USA). The human *cyclin D2* (*CCND2*) promoter (encompassing − 2329 to + 4 bp, where + 1 represents the transcription start site) was amplified by polymerase chain reaction (PCR) and subcloned into the pGL-3B vector (Promega, Madison, WT, USA). A series of 5ʹ-truncated promoter constructs and a *cyclin D2* promoter with mutation of a putative TCF4-binding site were also generated by conventional or inversed PCR-based methods. In addition, the human *EBP50* promoter encompassing − 1575 to − 70 bp was also subcloned into the pGL-3B vector (Promega) in a similar manner. The identity of all constructs was confirmed by sequencing prior to use. The sequences of PCR primers employed in this study are listed in Additional file 1: Table S1. The pcDNA-β-catenin (deleted S45), pCI-p300, pSG5-HEG0 (estrogen receptor (ER)-α), Glutathione S-transferase (GST)-fusion protein constructs including full length, PDZ1, PDZ2, and EB domains of EBP50, p3xFLAG-CMV14-EBP50, and TOP-reporter constructs were as described previously [15, 16].

Six Em Ca cell lines (Ishikawa, Hec6, Hec50B, Hec59, Hec88 and Hec265) were used as described previously [4]. The PTEN expression plasmid or empty vector was transfected into Hec6 cells (with a lack of endogenous PTEN expression due to the gene deletion) (Additional file 2: Fig. S1), and clones stably overexpressing (H6-PTEN) were established. EBP50-knockout line (H6-EBP-KO) was also generated using Hec6 cells (which have relatively high EBP50 expression). Briefly, guide RNA sequence (gRNA: 5ʹ-TCTATCTTCGCACTTTCCAC-3’) was designed using CRISPRdirect (https://crispr.dbcls.jp). The complementary oligonucleotides for gRNA were annealed and cloned into pSpCas9n(BB)-2A-Puro (PX462) V2.0 (Addgene #62,987). The pSpCas9n(BB)-2A-Puro (PX462) V2.0/gRNA construct was transfected into Hec6 cells and EBP-KO lines were also established. In H6-PTEN cells, spindle-shaped cells were defined as those that showed narrow and elongated phenotypes, along with weak or absent adhesion between cells. At least 200 cells were examined and the percentage with this morphology was reported.

### Antibodies and reagents

Antibodies used in this study are shown in Additional file 3: Table S2. Adriamycin (ADR: Catalog No. #D1515) was purchased from Sigma-Aldrich Chemicals (St. Louis, MO, USA).

### Transfection

Transfection was carried out using LipofectAMINE PLUS (Invitrogen), in duplicate or triplicate, in accordance with the manufacturer’s instructions. All reporter assays were carried out with 24-well plates and 0.4 µg of total plasmids. The pRL-TK plasmid (Promega) was used to normalize for transfection efficiency. Luciferase activity was assayed 24 h after transfection using the Dual-luciferase reporter assay system (Promega).

### Reverse transcription (RT)-PCR

cDNA was synthesized from 2 µg of total RNA. Amplification by RT-PCR was carried out in the exponential phase to allow comparison among cDNA synthesized from identical reactions using specific primers (Additional file 1: Table S1). Primers for the *GAPDH* gene were also used as described previously [17–19]. The intensity of individual signals was measured using ImageJ software version 1.41 (NIH, Bethesda, MD, USA). For quantitative analysis, real-time RT-PCR was also conducted using a Power SYBR Green PCR Master Mix (Applied Biosystems, Foster City, CA, USA). Fluorescent signals were detected using the ABI 7500 Real-time PCR System SDS Software (Applied Biosystems).

### Western blot and immunoprecipitation assays

Total cellular proteins were isolated using RIPA buffer [20 mM Tris–HCl (pH 7.2), 1% Nonidet P-40, 0.5% sodium deoxycholate, 0.1% sodium dodecyl sulfate]. Aliquots of the proteins were resolved by SDS-PAGE, transferred to membranes, and probed with primary antibodies, coupled with the ECL detection system (Amersham Pharmacia Biotechnology, Tokyo, Japan). For immunoprecipitation, cells were lysed with IP buffer [10 mM Tris–HCl (pH 7.6), 100 mM NaCl, 10% NP-40]. Cell lysates were cleared and incubated with anti-PTEN or anti-EBP50 antibodies, followed by incubation with Protein G-Sepharose (Amersham Pharmacia Biotechnology). Western blot assay was subsequently performed with anti-PTEN and anti-EBP50 antibodies.

### Flow cytometry and Aldefluor assay

Cells were fixed using 70% alcohol and stained with propidium iodide (Sigma) for cell cycle analysis. Aldehyde dehydrogenase 1 (ALDH1) enzyme activity in viable cells was determined using a fluorogenic dye-based Aldefluor assay (Stem Cell Technologies, Grenoble, France) according to the manufacturer’s instructions. The prepared cells were analyzed by flow cytometry using BD FACS Calibur (BD Biosciences) and CellQuest Pro software version 3.3 (BD Biosciences).

### Spheroid assay

Cells (× 10^3^) were plated in low cell binding plates (Thermo Fisher Scientific, Yokohama, Japan) in Cancer Stem Cell Premium (ProMab Biotech, Richmond, CA). Uniform spheroids of at least 50 µm in diameter were counted approximately two weeks after plating.

### Wound healing assay

Cells were seeded into 24-well tissue culture plates and grown to reach 90–100% confluence. After a cell monolayer formed, a wound was scratched with a sterile 200-µl tip. The area of the wound was also analyzed using ImageJ software version 1.41. Cell migration parameters were calculated in pixels as wound closure.

### RNA-seq assay

Total RNAs were extracted from CA-ALK and mock cells using the NucleoSpin RNA system (Takara). The concentration and quality of the RNA was verified with the Quantus Fluorometer (Promega) and Agilent 2100 Bioanalyzer, respectively. All the samples showed RIN values over 9. Total RNA (500 ng) was used for RNA library preparation, according to the instructions of the Quant Seq 3’ mRNA-Seq library prep kit FWD for Illumina (Lexogen, Vienna, Austria). The libraries were PCR-amplified for 12 cycles.

Sequencing of the libraries (via single-end 75-bp reads) was conducted on the Illumina NextSeq500 system. All data analyses were conducted using Strand NGS (v3.2, Agilent Technologies). The adapter sequences were removed from the raw reads, and base trimming was performed from the 3’ end of each read to remove bases with quality below Q10 up to a minimum length of 25 bp. Each read was mapped to the reference human genome hg38 with default settings. Expression patterns of transcripts were compared after normalization with DESeq [20] using default settings.

### GST pull-down assay

GST-EBP50-full length, GST-EBP50-PDZ1, GST-EBP50-PDZ2, and GST-EBP50-EB were induced by 1 mM isopropyl-β-D-thiogalactopyranoside and purified with glutathione-sepharose beads. Cell lysates were mixed with purified GST-EBP50-full length, GST-EBP50-PDZ1, GST-EBP50-PDZ2, or GST-EBP50-EB immobilized on the beads. Pull-down assays were performed at 4 °C overnight. The beads were then washed thoroughly with wash buffer [10 mM Tris–HCL (pH 7.5), 150 mM NaCl, 1 mM EDTA, and 1% Nonidet P-40]. Bound proteins were eluted by boiling in SDS-PAGE loading buffer, separated by SDS-PAGE, and detected by immunoblotting and Coomassie Brilliant Blue staining.

### Senescence-associated β-galactosidase (SA-β-gal) assay

Cells were stained for SA-β-gal activity as described previously [17]. At least 200 cells were evaluated for SA-β-gal staining and the labeling indices (LIs) were then calculated as a percentage.

### Clinical cases

A total of 102 cases of endometrioid-type Em Cas including 38 of grade (G)1, 33 of G2, and 31 of G3 were selected from the case records of Kitasato University Hospital during the period from 2007 to 2021, according to the criteria of the 2014 World Health Organization classification [21]. Of these, 38 cases of G1 or G2 Em Cas with pre-morular and morular lesions were observed. Pre-morule was defined as small morular lesions composed of less than 20 morular cells. All tissues were routinely fixed in 10% formalin and processed for embedding in paraffin. Approval for this study was given by the Ethics Committee of Kitasato University School of Medicine (B20-81).

### Immunohistochemistry (IHC)

IHC was performed using a combination of the microwave-oven heating and polymer immunocomplex (Envision, Dako) methods using whole sections. Briefly, after ordinary deparaffinization of 4-µm-thick sections, endogenous peroxidase was blocked by treatment of 0.3% hydrogen peroxide in methanol for 30 min. The microwave-oven heating was carried out with three 5-min cycles in either 10 mM citrate buffer (pH 6.0) or Tris buffer (pH 9.0). Routine IHC staining was then conducted using the polymer immunocomplex method. To assess the immunespecificity of each antibody, either normal mouse or rabbit sera was used as negative control instead of primary antibodies. Assessments of each sample (the scoring of IHC features) were made by three observers (AK, MM, and MS) and then compared.

For evaluation of IHC findings, scoring of cytoplasmic, membranous, or nuclear immunoreactivities in morular, premorular, and the surrounding carcinoma (Sur Ca) components, respectively, was performed on the basis of the percentage of immunopositive cells and the immunointensity with multiplication of the values of the two parameters as described previously [17–19]. Nuclear Ki-67 immunopositivity was also counted in at least 200 cells from the three lesions, respectively, and the LIs were then calculated as a percentage.

### Immunofluorescence

The slides were heated in 10 mM citrate buffer (pH 6.0) for 3 × 5-min cycles using a microwave oven and then incubated overnight with anti-PTEN, anti-β-catenin, or anti-EBP50 antibodies. Alexa 488 and 570 (Thermo Fisher Scientific, Waltham, MA, USA) were used as secondary antibodies.

### Mutation analyses of the PTEN gene

Genomic DNA was extracted from Hec6 cells using a Wizard Genomic DNA Purification kit (Promega) according to the manufacturer’s instructions. Exons 1 to 9 of the *PTEN* gene were amplified by PCR and the products were subsequently subjected to direct sequencing PCR as described previously [18]. The sequences of primers used in this study are listed in Additional file 1: Table S1.

### Methylation analysis of the cyclin D2 promoter

Genomic DNA extracted from cell lines using a Wizard Genomic DNA Purification kit (Promega) was treated by bisulfate using an EZ DNA Methylation-Gold kit (ZYMO Research, Orange, CA, USA). Bisulfate-treated DNA was amplified by PCR using specific primers for the *cyclin D2* promoter and the methylation status was analyzed as described previously [22].

### RNAscope assay for PTEN and cyclin D2 mRNA in situ hybridization (ISH)

Expression of PTEN and cyclin D2 mRNA was analyzed using an RNAscope assay (Advanced Cell Diagnostics, Hayward, CA, USA) according to manufacturer’s instructions. The hybridization was performed with targeted probes: Hs-PTEN (#408,511), Hs-CCND2 (cyclin D2)(#470,031), positive control probe (#2,010,684), and negative control probe (#310,043) for 2 h at 40 °C. Numbers of intracytoplasmic ISH signals were counted in at least 50 cells and were then expressed as an average number of signals per cell.

### Statistics

Comparative data were analyzed using the Mann–Whitney *U*-test and Spearman’s correlation coefficient. The cut-off for statistical significance was set as *p* < 0.05.

## Results

### PTEN overexpression leads to induction of EMT features, decreased cell proliferation, and increased apoptosis in Em Ca cells

**Fig. 1:**
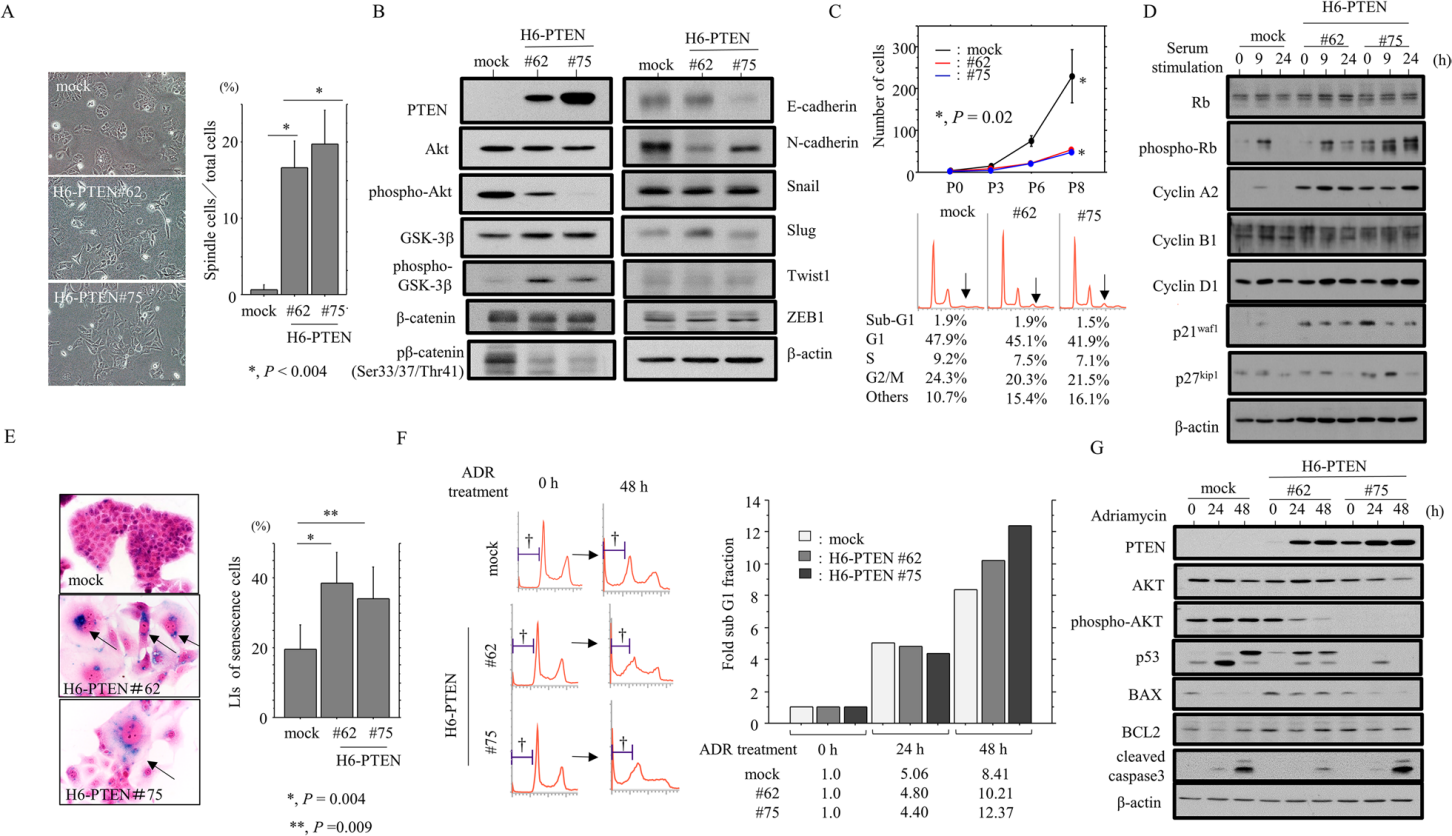
Changes in phenotypic characteristics of Em Ca cells stably overexpressing PTEN. **A** Left: phase contrast images of H6-PTEN cells. Note the changes in cell morphology toward fibroblastic appearances in H6-PTEN cells. Right: the numbers of spindle-shaped cells are presented as means ± SDs. **B** Western blot analysis for the indicated proteins in total lysates from H6-PTEN and mock cells. **C** Upper: two independent H6-PTEN and mock cell lines were seeded at low density. The cell numbers are presented as means ± SDs. P0, P3, P6, and P8 are 0, 3, 6, and 8 days after seeding, respectively. Lower: flow cytometry analysis of H6-PTEN and mock cells 3 days after seeding (P3). **D** Western blot analysis for the indicated proteins in total lysates from H6-PTEN and the mock cells following re-stimulation of serum-starved (24 h) cells with 10% serum for the indicated times. **E** Left: SA-β-gal assay for H6-PTEN and mock cells. Note the blue dot aggregates in the cytoplasm of senescent-like cells (indicated by arrows). Original magnification, × 400. Right: labeling indices for SA-β-gal positive cells are demonstrated as a percentage. **F** Left: after treatment of H6-PTEN and mock cells with 1 µg/mL Adriamycin (ADR) from the time shown, cell undergoing apoptosis (sub-G1) were detected by flow cytometry. Daggers indicate the sub-G1 fraction. Right: the fold sub-G1 fractions were calculated. The sub-G1 fraction values in 0 h were set as 1. **G** Western blot analysis for the indicated proteins in total lysates from H6-PTEN and mock cells treated with 1 µg/mL ADR for the times shown

To examine the functional role of PTEN overexpression in Em Ca cells, we first established two independent cell lines in which PTEN was exogenously overexpressed (H6-PTEN #62 and #75) using Hec6 cells with a lack of endogenous PTEN expression due to the gene deletion (Additional file 2: Fig. S1). The H6-PTEN cells demonstrated a significant switch towards a fibroblastic morphology (Fig. 1A), along with increased expression of phospho-GSK-3β and Slug, and decreased expression of phospho-AKT, phospho-β-catenin, E-cadherin, and N-cadherin (Fig. 1B).

To examine whether PTEN overexpression affects cell proliferation, the two independent H6-PTEN cell lines were seeded at low density. H6-PTEN cells tended to proliferate more slowly, particularly in the exponential growth phase, along with a decreased proportion of cells in G1, S, and G2/M phase of the cell cycle; this was in contrast to the increased fraction of cells with > 4n DNA (Fig. 1C). To further examine alterations in the expression of several cell cycle-related molecules during cell growth, the H6-PTEN cells were rendered quiescent by serum starvation and were subsequently stimulated with serum. At 9 h, and 24 h after release into the cell cycle, the expression of phospho-Rb, Cyclin A2, p21^waf1^, and p27^kip1^ was substantially increased in H6-PTEN cells relative to the mock cells (Fig. 1D). In addition, a significant increase in the number of SA-β-gal positive cells was observed in the stable lines (Fig. 1E).

Next, we examined the association between PTEN overexpression and apoptotic features in response to a cytotoxic stimulus. Treatment of H6-PTEN cells with ADR increased subG1 fractions as compared to the mock (Fig. 1F), along with decreased phospho-AKT and increased BAX expression (Fig. 1G).

These findings suggest that PTEN overexpression contributes to induction of EMT-like features; these changes are accompanied by inhibition of cell proliferation, increased senescence features, and susceptibility to apoptosis.

### PTEN overexpression is associated with acceleration of cell mobility and CSC-like features

**Fig. 2:**
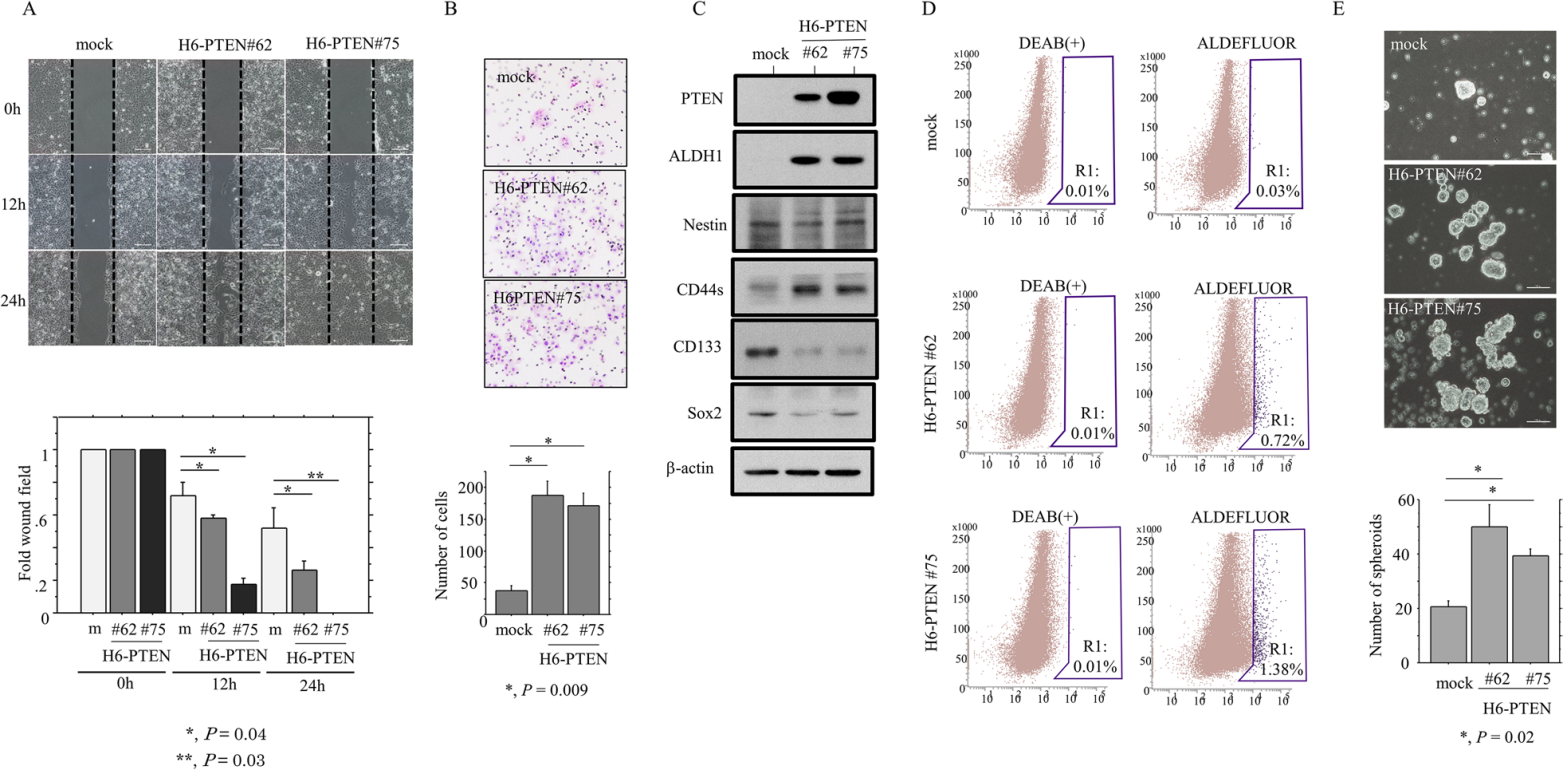
Relationship between PTEN overexpression, cell migration, and cancer stem cell-like features. **A** Upper: wound-healing assay with H6-PTEN and mock cells. A scratch ‘wound’ was introduced to the middle of wells containing cells grown to confluency, and phase contrast images were taken after 12 and 24 h. Lower: the values of wound areas in 0 h were set as 1. The fold wound areas are presented as means ± SDs. **B** Migration rate measured using a transwell assay. Upper: the H6-PTEN and mock cells were seeded in a 24-well transwell plates and incubated for 24 h in medium without serum. Cells were stained with HE and counted using a light microscope. Lower: the numbers of migrated cells are presented as means ± SDs. **C** Western blot analysis for the indicated proteins in total lysates from H6-PTEN and mock cells. **D** Aldefluor analysis in H6-PTEN and mock cells. Note the R1 populations including the ALDH^high^ population with cancer stem cell-like features. **E** Upper: phase-contrast photographs of spheroids formed by H6-PTEN and mock cells following 2 weeks of growth. Lower: the numbers of spheroids are presented as means ± SDs

To examine whether PTEN overexpression contributes to cell motility through EMT-like features, we carried out scratch and migration assays. The H6-PTEN cells refilled wounded empty spaces more rapidly (Fig. 2A), in line with the significantly increased migration rates as compared to the mock cells (Fig. 2B).

Since EMT also promotes stem cell properties and further generates cells with cancer stem cell (CSC)-like features [23], we examined the association between PTEN overexpression and CSC-like properties. H6-PTEN cells demonstrated an increased expression of ALDH1 and CD44s, in contrast to decreased CD133 and Sox2 expression (Fig. 2C). There was a significant increase in the ALDH^high^ population, which includes a high percentage of CSC-like cells, in the H6-PTEN cells compared to the mock cells (Fig. 2D). This was in line with the significant increase in the number of well-defined, round spheroids > 50 µm in diameter (Fig. 2E).

These findings suggest that PTEN overexpression engenders CSC-like features and accelerates cell motility in Em Ca cells.

### Upregulation of cyclin D2 mediated by β-catenin in cells overexpressing PTEN

**Fig. 3:**
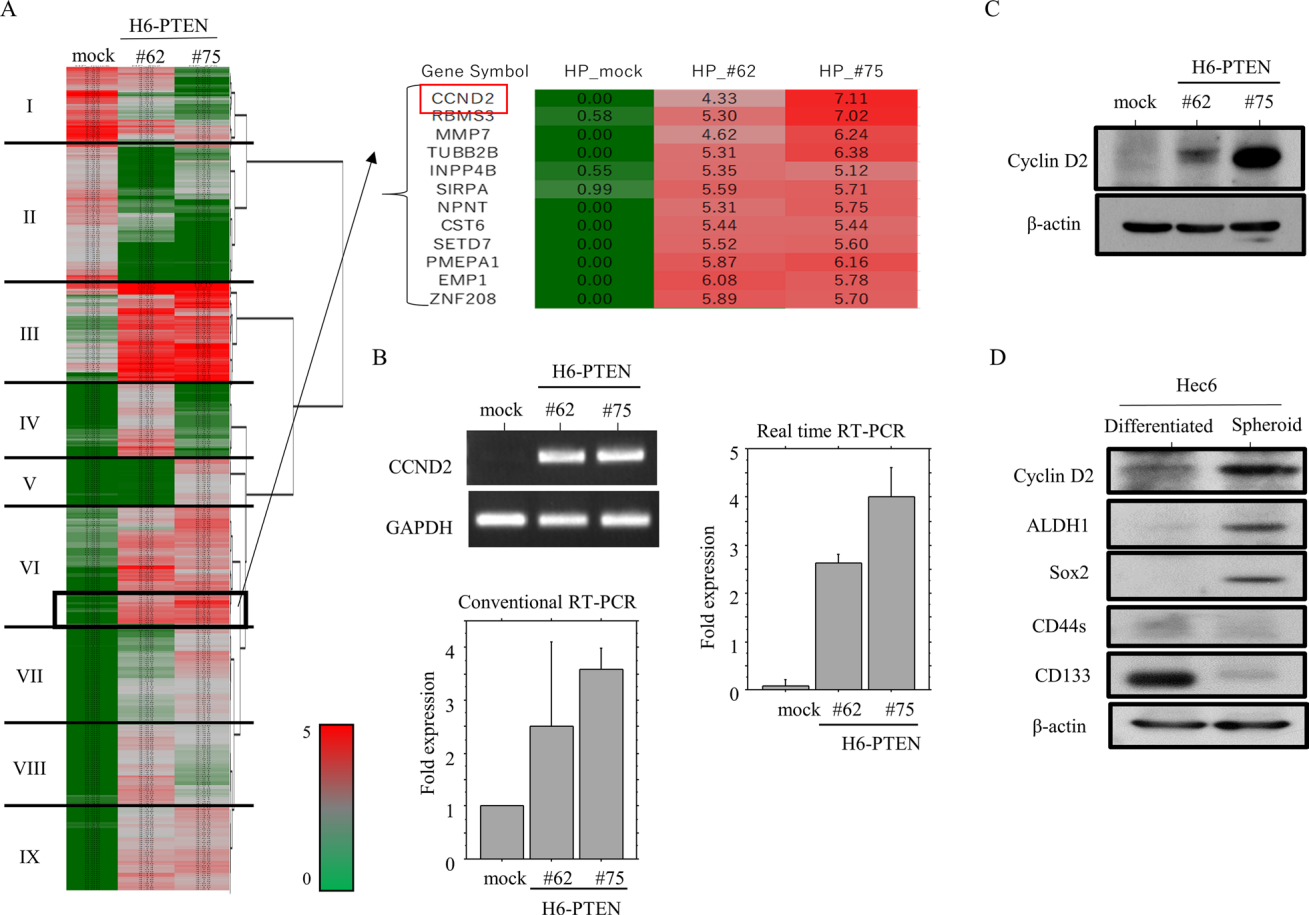
Relationship between PTEN overexpression and cyclin D2 expression. **A** Unsupervised hierarchical clustering of mRNA expression detected by RNA-seq assay in H6-PTEN and mock cells. The level of expression of each mRNA is colored; red, black, and green indicated high (> 4), neutral (1–4), and low (< 1), respectively. Major clusters are shown as groups I to IX. *Cyclin D2 (CCND2)* is included in group VI. **B** Analysis of endogenous cyclin D2 mRNA expression by conventional (left upper and lower) and real time RT-PCR assay (right) in H6-PTEN and mock cells. The values of endogenous cyclin D2 mRNA expression detected by conventional RT-PCR assay were normalization to GAPDH. The fold changes in mRNA expression for both assays are presented as means ± SDs (left lower). **C** Western blot analysis for the indicated proteins in total lysates from H6-PTEN and mock cells. **D** Western blot analysis for the indicated proteins in total lysates from spheroid and differentiated Hec6 cells

To identify genes that are differentially expressed following PTEN overexpression, RNA-seq assays were carried out using total RNAs extracted from H6-PTEN cells. A total of 7164 and 7182 genes in H6-PTEN#62 and #75 cells were dysregulated, respectively. Of these, 251 and 331 genes were upregulated or downregulated over fivefold, respectively, in the H6-PTEN cells as compared to the mock cells. Hierarchical clustering revealed that the genes could be readily categorized into nine groups. We focused on the *cyclin D2 (CCND2)* gene in group VI that was overexpressed by 4 to sevenfold (Fig. 3A). This is because cyclin D2 is required for persistent CSC growth via the maintenance of an intact cell cycle and proliferation [24]. Cyclin D2 mRNA and protein expression were clearly increased in H6-PTEN cells (Fig. 3B, C). Expression of cyclin D2, as well as ALDH1 and Sox2, were clearly increased in Hec6-spheroid cells (CSC) as compared to the differentiated Hec6 cells (Fig. 3D). There were no changes in the methylation status of the *cyclin D2* promoter in H6-PTEN compared to mock cells (Additional file 4: Fig. S2).

**Fig. 4:**
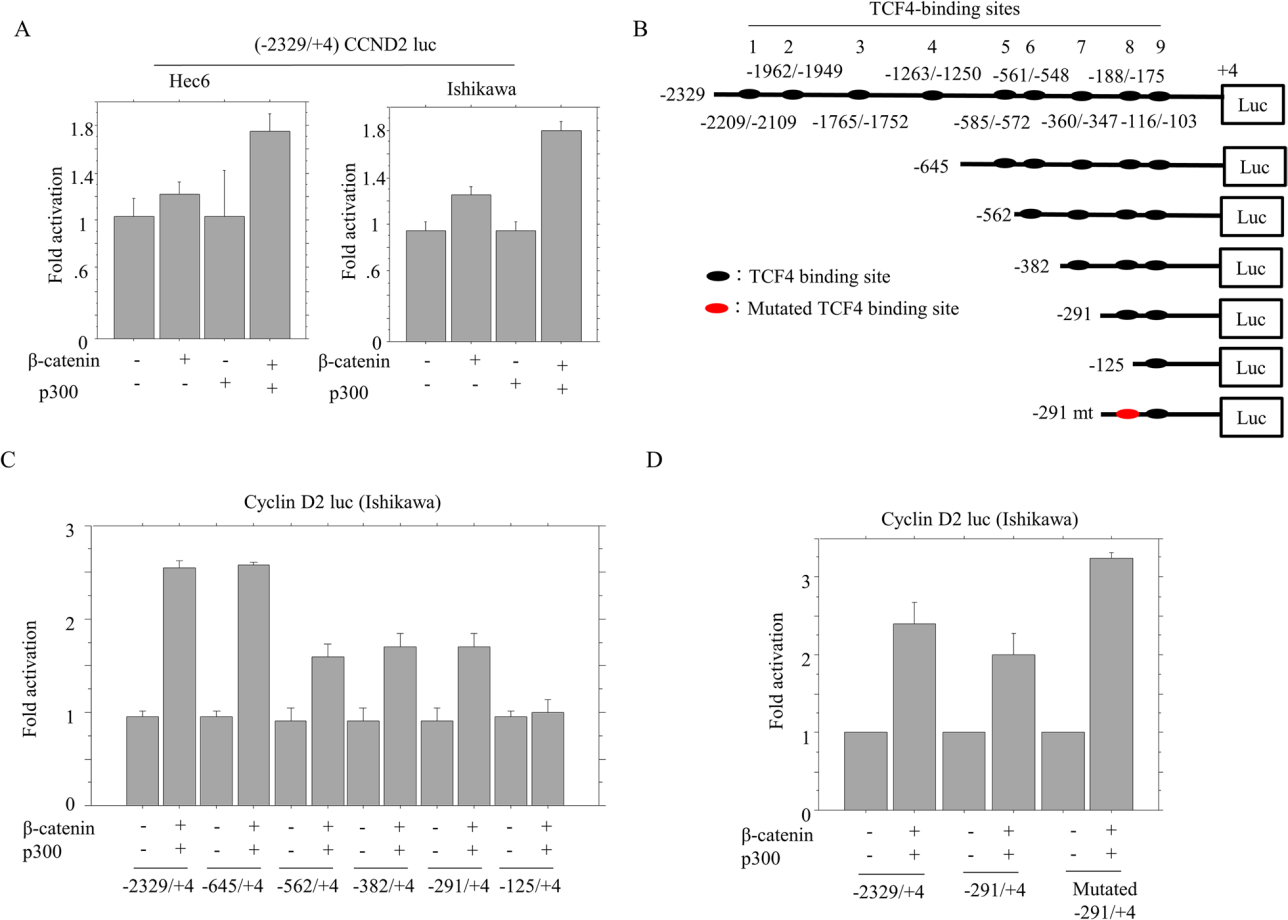
Transcriptional regulation of cyclin D2 expression by β-catenin/p300 in Em Ca cells. **A** Hec6 and Ishikawa cells were transfected with cyclin D2 reporter constructs, together with β-catenin and p300. Relative activity was determined based on arbitrary light units of luciferase activity normalized to pRL-TK activity. The activities of the reporter plus the effector relative to that of the reporter plus empty vector are shown as means ± SDs. **B** Various promoter deletion constructs used for evaluating transcriptional regulation of the *cyclin D2* promoter by β-catenin/p300. Note the nine TCF4-binding elements in the promoter region. Results of introduction of mutations into − 291/ + 4-cyclin D2-Luc are also demonstrated. **C** Ishikawa cells were transfected with various 5ʹ-truncated constructs of the *cyclin D2* promoter, along with β-catenin/p300. **D** Ishikawa cells were transfected with two wild-type *cyclin D2* promoters and the mutated form, along with β-catenin/p300

Cyclin D2 is normally expressed at the base of the intestinal crypt, where there are the highest levels of Wnt signaling [25]. We further asked whether the *cyclin D2* promoter is affected by β-catenin status, since phospho-β-catenin (the inactive form) was decreased in H6-PTEN cells. Co-transfection of β-catenin and p300 could activate *cyclin D2* promoter about 1.8 fold in both Hec6 and Ishikawa cells (Fig. 4A). A search of the *cyclin D2* promoter for a potential TCF4-binding site (TBS, CTTTG T/A T/A) revealed the presence of nine sites (Fig. 4B). In a series of 5ʹ-truncated promoter constructs (Fig. 4B), the deletion from − 2329 to − 291 bp had little effect on induction of promoter activity by a combination of β-catenin and p300. In contrast, deletion of − 291 to − 125 bp reduced in the responsiveness, indicating that β-catenin/p300-responsive elements might be located in this region, which includes the TBS-8 site (Fig. 4C). However, β-catenin-dependent transactivation was not affected by introduction of 12-nucleotide alterations in the TBE-8 site (Fig. 4D).

These findings suggest that upregulation of *cyclin D2* mediated by β-catenin contributes to the CSC-like features associated with PTEN overexpression in Em Ca cells.

### PTEN overexpression in morular but not the surrounding carcinoma lesions of Em Ca tissues

Strong cytoplasmic PTEN immunoreactivity was frequently observed in all morular components in 38 cases of Em Ca with morules (Additional file 5: Fig. S3A and Additional file 6: Table S3), in contrast to the relatively weak immunoreaction in the Sur Ca. The mRNA signals were also opposite in the two lesion types (Additional file 5: Fig. S3B). Specifically, PTEN immunopositivity in the Sur Ca appeared to be higher in Em Ca with morules as compared to the tumors lacking such lesions, although the difference did not reach the set level of significance (Additional file 6: Table S3).

**Fig. 5:**
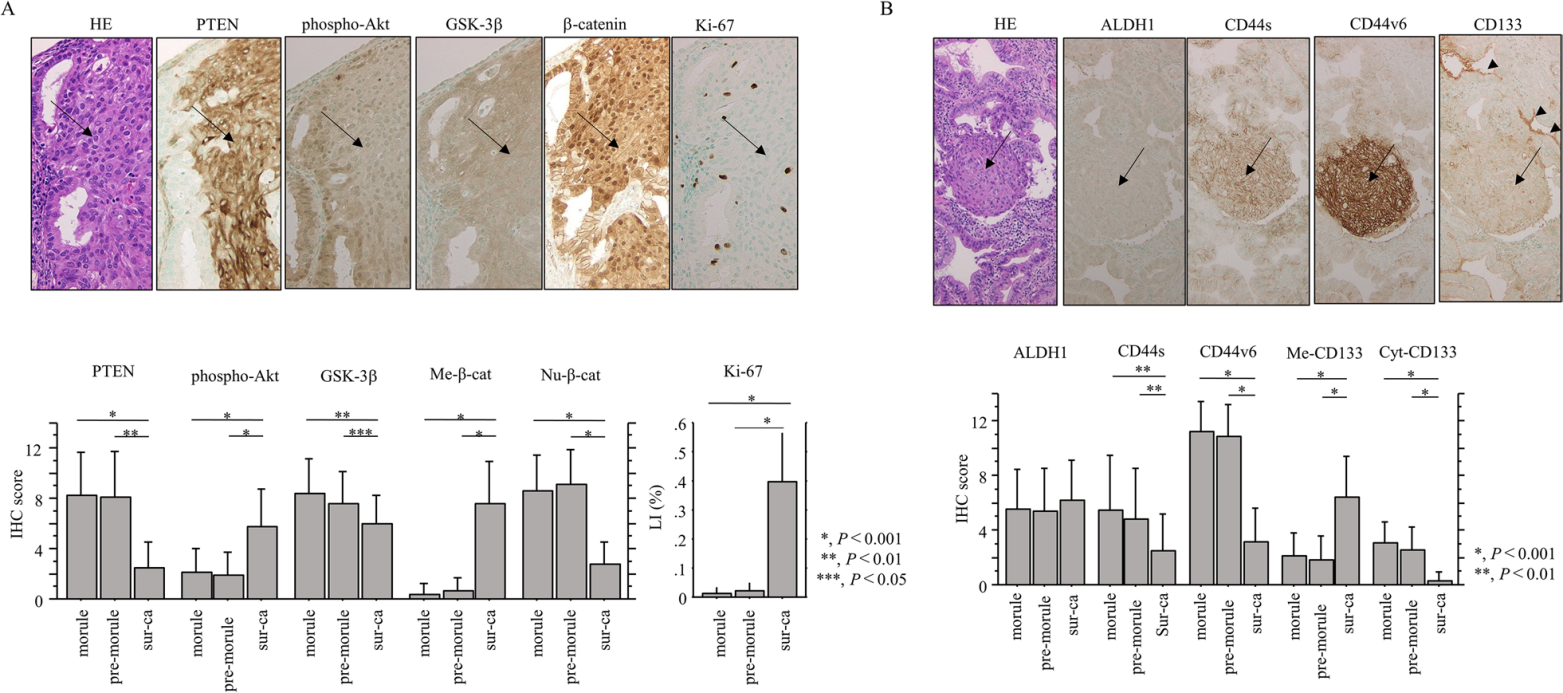
IHC findings in serial sections of Em Ca with morules. **A**, **B** Upper: HE and IHC staining for the indicated proteins in Em Ca with morule. Morular lesions and membranous CD133 immunoreactivity are indicated by arrows and arrowheads, respectively. Original magnification, × 200. Lower: IHC score or LIs for the indicated molecules in Em Ca with morules. The data shown are means ± SDs. Me-β-cat, membranous β-catenin; Nu-β-cat, nuclear β-catenin; Me-CD133, membranous CD133; Cyt-CD133, cytoplasmic CD133

Representative IHC findings for PTEN and related markers, as well as CSC-related molecules, in Em Ca with morules and pre-morules are illustrated in Fig. 5 and Additional file 7: Fig. S4A and B. IHC scores for PTEN, GSK-3β, and nuclear β-catenin, as well as CD44S, CD44v6, and cytoplasmic CD133, were significantly higher in morular and pre-morular lesions as compared to those of the Sur Cas of Em Ca. In contrast, phospho-AKT, membranous β-catenin and membranous CD133 scores, and Ki-67LIs were significantly higher in the latter (Fig. 5). PTEN score was positively correlated with nuclear β-catenin, CD44v6, and cytoplasmic CD133 scores, and inversely with membranous β-catenin score and Ki-67 LIs in Em Ca with morules (Table 1).

**Table 1:** Correlation between expression of PTEN and related molecules in endometrial carcinoma with morules

	Me-EBP50	ERα	Me-β‐catenin	Nu-β‐catenin	PTEN	Cyt/Nu-pAKT	Cyt/Nu-GSK3β	Ki-67	CD44s	CD44v6	Me-CD133
	*ρ*(*P*)	*ρ* (*P*)	*ρ* (*P*)	*ρ* (*P*)	*ρ* (*P*)	*ρ* (*P*)	*ρ* (*P*)	*ρ* (*P*)	*ρ* (*P*)	*ρ* (*P*)	*ρ* (*P*)
ERα	0.80 (< 0.0001)	*	*	*	*	*	*	*	*	*	*
Me- β‐catenin	0.73 (< 0.0001)	0.73 (< 0.0001)	*	*	*	*	*	*	*	*	*
Nu-β‐catenin	− 0.75 (< 0.0001)	− 0.71 (< 0.0001)	− 0.67 (< 0.0001)	*	*	*	*	*	*	*	*
Cyclin D1	− 0.21 (0.02)	− 0.30 (0.001)	− 0.33 (0.0004)	0.25 (0.006)	*	*	*	*	*	*	*
PTEN	− 0.63 (< 0.0001)	− 0.58 (< 0.0001)	− 0.62 (< 0.0001)	0.49 (< 0.0001)	*	*	*	*	*	*	*
Cyt/Nu-pAKT	0.62 (< 0.0001)	0.56 (< 0.0001)	0.48 (< 0.0001)	− 0.40 (< 0.0001)	− 0.24 (0.008)	*	*	*	*	*	*
Cyt/Nu-GSK3β	− 0.34 (0.0003)	− 0.23 (0.0131)	− 0.17 (0.06)	0.35 (0.0002)	0.07 (0.4)	− 0.07 (0.4)	*	*	*	*	*
Ki-67	− 0.30 (0.001)	0.76 (< 0.0001)	0.69 (< 0.0001)	0.69 (< 0.0001)	− 0.60 (< 0.0001)	0.53 (< 0.0001)	− 0.14 (0.1)	*	*	*	*
CD44s	− 0.35 (0.0002)	− 0.28 (0.002)	− 0.13 (0.1)	− 0.13 (0.1)	0.09 (0.3)	− 0.2 (0.002)	0.29 (0.001)	− 0.20 (0.03)	*	*	*
CD44v6	− 0.79 (< 0.0001)	− 0.72 (< 0.0001)	− 0.69 (< 0.0001)	− 0.69 (< 0.0001)	0.51 (< 0.0001)	− 0.51 (< 0.0001)	0.37 (< 0.0001)	− 0.64 (< 0.0001)	0.48 (< 0.0001)	*	*
Me-CD133	0.60 (< 0.0001)	0.66 (< 0.0001)	0.58 (< 0.0001)	0.58 (< 0.0001)	− 0.41 (< 0.0001)	0.45 (< 0.0001)	− 0.11 (0.2)	0.59 (< 0.0001)	− 0.26 (0.005)	− 0.55 (< 0.0001)	*
Cyt-CD133	− 0.68 (< 0.0001)	− 0.61 (< 0.0001)	− 0.55 (< 0.0001)	− 0.55 (< 0.0001)	0.45 (< 0.0001)	− 0.43 (< 0.0001)	0.28 (0.002)	− 0.63 (< 0.0001)	0.23 (0.01)	0.65 (< 0.0001)	− 0.24 (0.009)

* Not examined

*ρ* Spearman’s correlation coefficient; *Me* Membrane; *Nu* Nuclear; *Cyt* Cytoplasmic

As shown in Additional file 5: Fig. S3C, cyclin D2 mRNA signals were detected in both morular and Sur Ca components, but there was no difference in the signal numbers between the two lesions.

These findings suggest that PTEN-post-translational stabilization and nuclear β-catenin accumulation are early events in Em Ca during trans-differentiation towards the morular phenotype with CSC-like features.

### EBP50 is negatively associated with PTEN and β-catenin expression in Em Ca

**Fig. 6:**
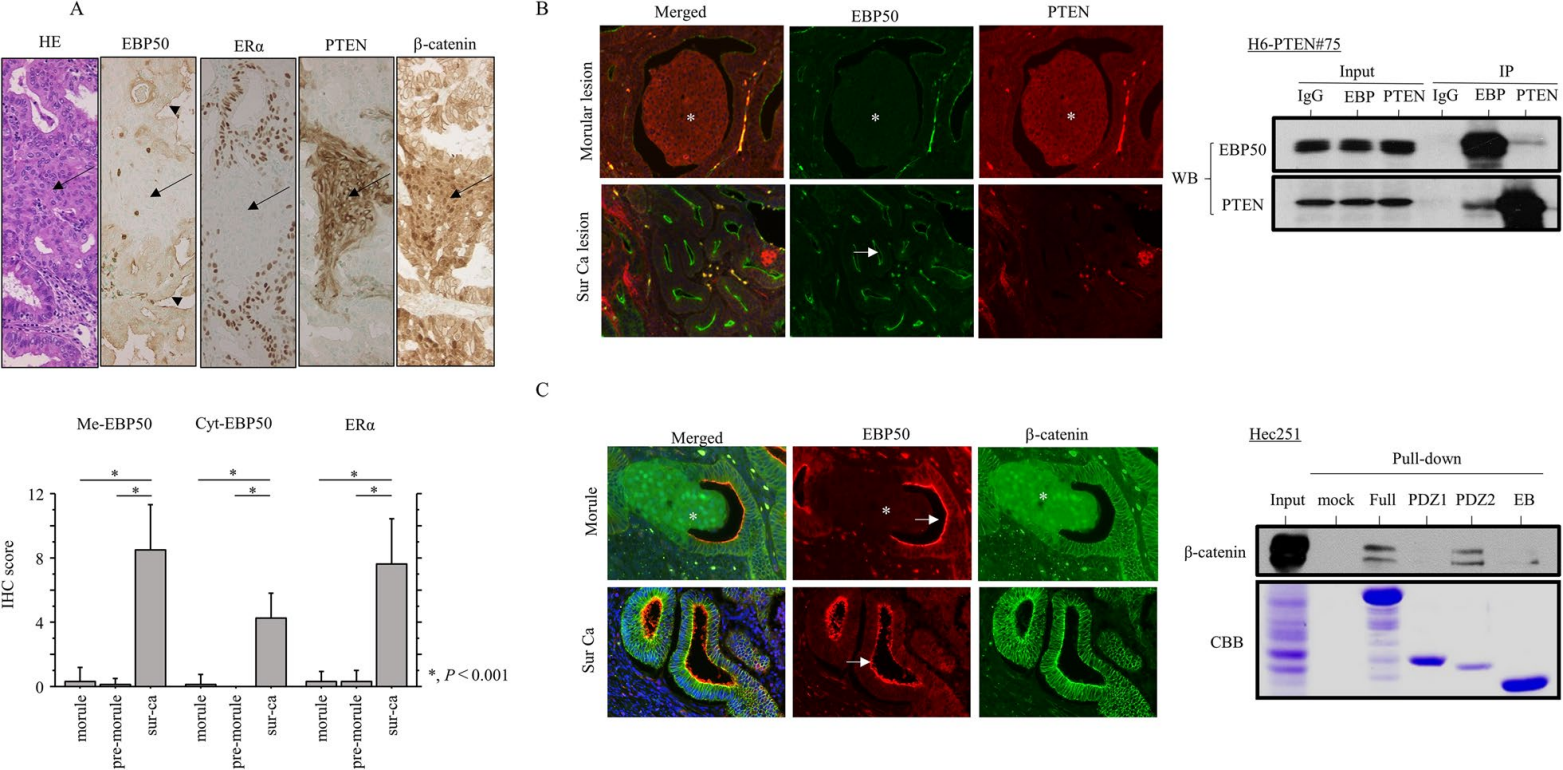
Relationship between EBP50, PTEN, and β-catenin in Em Ca with morules. **A** Upper: HE and IHC staining for the indicated proteins in Em Ca with morules. Morular lesions and membranous EBP50 immunoreactivity are indicated by arrows and arrowheads, respectively. Original magnification, × 200. Lower: IHC score for the indicated molecules in Em Ca with morules. The data shown are as means ± SDs. Me-EBP50, membranous EBP50; Cyt-EBP50, cytoplasmic EBP50. **B** Left: immunofluorescence for EBP50 and PTEN. A lack of coimmunolocalization for EBP50 and PTEN in morules (indicated by asterisks) and the surrounding carcinoma (Sur Ca, indicated by an arrow) in Em Ca with morules. Original magnification, × 200. Right: western blotting (WB) with anti-EBP50 (upper panel) and anti-PTEN antibodies (lower panel) after immunoprecipitation (IP) with the indicated antibodies using H6-PTEN cell lysates. Input represents 5% of the total cell extract. Normal rabbit IgG was used as a negative control. **C** Left: immunofluorescence for EBP50 and β-catenin. Note the coimmunolocalization of EBP50 and β-catenin in the apical membrane of Sur Ca cells (indicated by arrows) but not morules (indicated by asterisks). Original magnification, × 200. Right: proteins bound to the beads were analyzed following by western blot analysis for β-catenin in Hec251 cells (upper). Detection of GST-bound EBP50 protein by Coomassie Brilliant Blue (CBB) (lower)

EBP50 is an adaptor protein required for epithelial morphogenesis and is regulated by estrogen [13]. Since EBP50 is able to interact with both PTEN and β-catenin [13], we asked whether there was an association of EBP50 with the two molecules. Representative IHC findings for EBP50, ERα, PTEN, and β-catenin in Em Ca with morules and premorules are illustrated in Fig. 6A and Additional file 7: Fig. S4C. Cytoplasmic or membranous EBP50 and nuclear ERα scores were significantly higher in Sur Cas as compared to those of morular lesions in Em Ca. EBP50 score was positively correlated with ERα score; this is consistent with our observations that the *EBP50* promoter was activated by ERα in a dose-dependent manner (Additional file 8: Fig. S5A), whereas there was an inverse association of EBP50 with both PTEN and nuclear β-catenin status (Table 1). The latter was consistent with the EBP50-dependent inhibition of β-catenin-mediated TOP activity in Em Ca cells (Additional file 8: Figure S5B).

Coimmunolocalization of PTEN and EBP50 was not observed in Sur Ca lesions, although the association between the two was confirmed by immunoprecipitation-western blot analysis (Fig. 6B). In contrast, colocalization of β-catenin and EBP50 was evident in apical membranes of glandular but not morular lesions, in line with specific binding of β-catenin to the GST-EBP50-PDZ2 domain (Fig. 6C).

Finally, we established two independent EBP50-knockout lines (H6-EBP-KO) using Hec6 cells. The H6-EBP-KO cells demonstrated more spread and flattened features (Additional file 9: Fig. 6A), along with an increased expression of β-catenin and phospho-GSK-3β and decreased phospho-β-catenin expression as compared to those of the mock cells (Additional file 9: Fig. 6B).

These findings suggest that loss of ERα/EBP50 contributes to PTEN overexpression and nuclear β-catenin stabilization in morular lesions of Em Ca.

## Discussion

The present study clearly provides evidence that Em Ca cells stably overexpressing PTEN (H6-PTEN) exhibit EMT-like features, probably through β-catenin/Slug-meditated E-cadherin suppression [26]; this likely underlies the enhancement of migration capability. PTEN overexpression also inhibited cell proliferation, accelerated cellular senescence, and increased apoptotic features, along with upregulation of p21^waf1^, p27^kip1^, and BAX expression; these observations are consistent with the inverse correlation between cell proliferation and migration [27–29]. Given that EMT involves loss of epithelial polarity, a migratory phenotype, and a switch to a mesenchymal-like program [30, 31], we suggested that PTEN overexpression contributes to changes in the phenotypic characteristics of Em Ca cells due to induction of EMT-like features. In contrast, PTEN *inactivation* engenders EMT phenotypes in breast, lung, and colorectal carcinomas [32–34].

We also found that H6-PTEN cells had significant CSC-like properties, along with high expression of some stemness markers, a large ALDH1^high^ population, and enriched spheroid formation. In addition, the stable cells also increased mRNA and protein expression of cyclin D2, which is required for persistent CSC growth through the maintenance of an intact cell cycle and proliferation with low DNA damage accumulation [24, 35]. Given that PTEN is critical for stem cell maintenance and that its loss can lead to the emergence and proliferation of CSC clones [36, 37], we suggest that PTEN overexpression may also play an important role in establishment and maintenance of CSC-like properties in Em Ca cells. This would be consistent with the evidence of a close association between EMT features and CSC properties [23]. However, PTEN deficiency leads to upregulation of PAX7, which in turn promotes oncogenic transformation of human neural stem cells and confers aggressiveness to human glioblastoma stem cells [38]. Thus, the functional role of PTEN may be highly contextual, and dependent on both tissue of origin and cell type.

An interesting finding in this study is that strong PTEN immunoreactivity was observed in all morular components, despite the lower levels in Sur Ca lesions. Moreover, PTEN immunopositivity in Sur Ca was higher in Em Ca with morules when compared to tumors lacking such lesions. Given that both Sur Ca and morular elements are integral components of a common progenitor cell in Em Ca [11, 39], we suggest that PTEN mutations may be rare in cases of Em Ca with morules. In fact, the frequency of PTEN inactivation in morule-containing endometrial intraepithelial lesions (13%, 1/8) is significantly lower compared with the 63% (22/35) inactivation reported previously for all EIN lesions [40].

Several lines of evidence from the present study support the conclusion that cyclin D2 is transcriptionally regulated by nuclear β-catenin. First, the expression of both β-catenin and cyclin D2 at the mRNA and protein levels was increased in H6-PTEN cells. Second, the expression of cyclin D2 mRNA coincided with nuclear β-catenin stabilization and PTEN overexpression in morular lesions of Em Ca, and this was independent of cell proliferation. Third, *cyclin D2* promoter was directly or indirectly activated by cotransfection of β-catenin/p300.

More importantly, immunoreactivities for PTEN, as well as CSC-related molecules, were significantly higher in both morular and premorular lesions as compared to the Sur Ca of Em Ca, and were positively correlated with nuclear β-catenin stabilization and negatively correlated with cell proliferation. Given that β-catenin expression was increased in H6-PTEN cells due to inactivation of GSK-3β, we suggest that a combination of PTEN and β-catenin may act as an inducer of morular formation in Em Ca cells. This would in turn lead to EMT/CSC-like properties accompanied with upregulation of cyclin D2. Our conclusion is supported by the finding that morules represent foci of very early squamous differentiation and are precursors of typical squamous elements [41].

Although phospho-AKT can inhibit GSK-3β activity through phosphorylation of the Ser9 site [42], we unexpectedly observed that levels of phospho-GSK-3β (the inactive form) were increased in H6-PTEN cells, despite the lower phospho-AKT (the active form), and this led to decreased phosphorylation of β-catenin at Ser33/37/Thr41 (the form that is primed for degradation). Given that phosphorylation of Ser9-GSK-3β can be mediated by a large number of different kinases [43], an AKT-independent mechanism may contribute to the GSK-3β phosphorylation in H6-PTEN cells. For example, GSK-3β activity can be modulated either by growth factors that work through the PI3K-protein kinase B cascade or by hormonal stimulation of G protein-coupled receptors that are linked to changes in intracellular cAMP levels [44].

Finally, we have provided the first evidence that EBP50 acts as a negative regulator for morular formation of Em Ca cells, as follows. First, immunoreactivities for both EBP50 and ERα were reduced or absent in morules as compared to Sur Ca; this is consistent with our observation that ERα could activate the *EBP50* promoter in a dose-dependent manner. Second, coimmunoprecipitation of EBP50 and PTEN was observed in Em Ca cell lines, but we failed to observe their colocalization in Em Ca tissues, probably due to the low PTEN immunoreactivity in Sur Cas. Interestingly, the finding of significantly lower PTEN mRNA expression in morules when compared to Sur Ca indicates the existence of post-translational PTEN regulation in the former. Third, EBP50 also interacted with β-catenin through its PDZ2 domain at the apical cell membrane of cells in Sur Ca but not in morules, leading to an inhibition of β-catenin-dependent transcriptional activity. Finally, knockout of EBP50 stabilized β-catenin without altering markers of AKT/GSK-3β signaling. These findings suggest that EBP50 depletion may be due to membrane displacement of PTEN and nuclear translocation of excess β-catenin.

## Conclusion

**Fig. 7:**
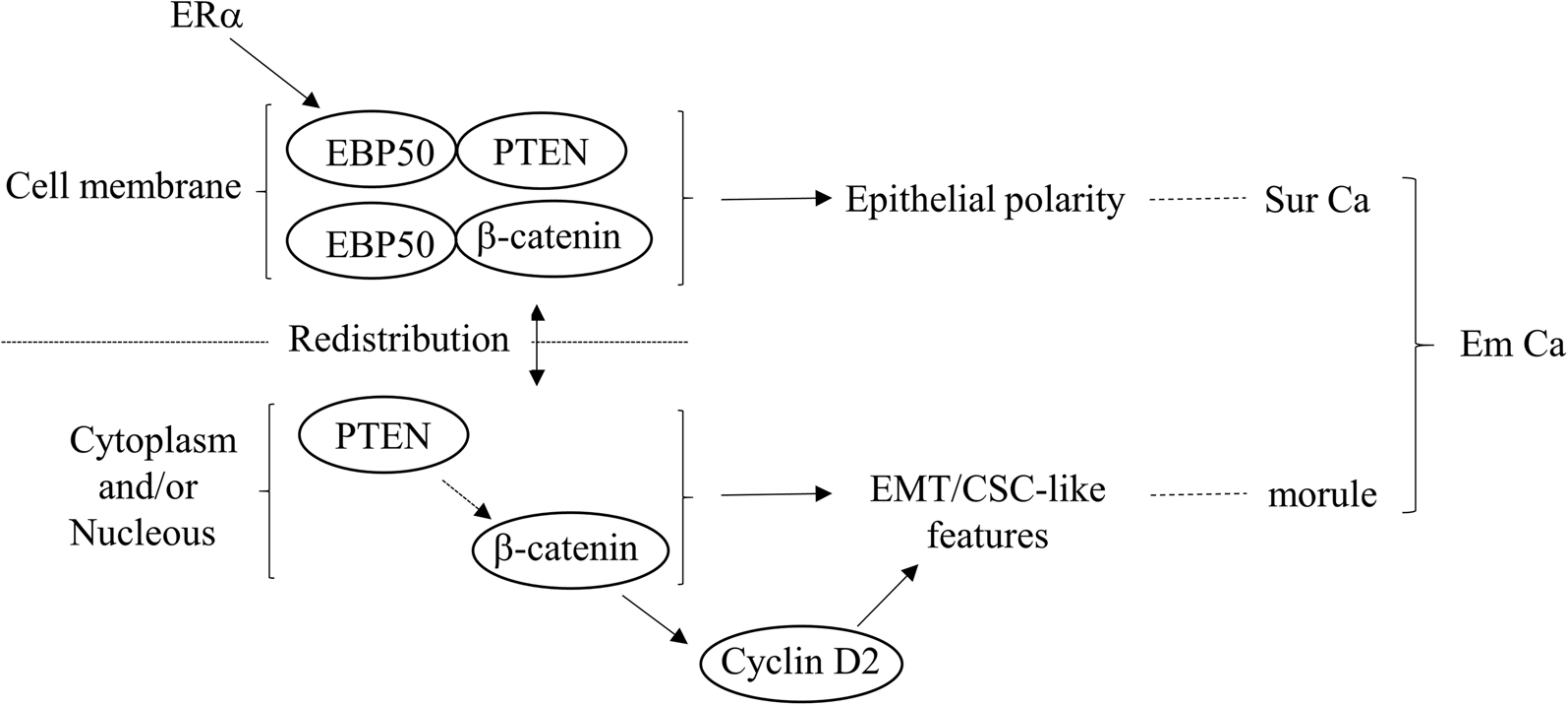
Schematic representation of the interplay between the EBP50, PTEN, and β-catenin during *trans*-differentiation towards the morular phenotype associated with EMT/CSC-like features in Em Ca cells

Our observations suggest a model for the functional role of PTEN overexpression and nuclear β-catenin stabilization during morular formation in Em Ca cells (Fig. 7). Loss of ERα/EBP50 may be an initial signal for disruption of apical-basal polarity and subcellular redistribution of PTEN and β-catenin; this in turn may lead to establishment and maintenance of the morular phenotype associated with EMT/CSC-like features in Em Ca cells.

## Supplementary Information

**Additional file 1: Table S1.** Primer sequences used in this study

**Additional file 2: Fig. S1.** PTEN expression and mutation in Hec6 cells. Upper: western blot analysis for PTEN proteins in total lysates from six Em Ca cell lines. Lower: sequencing analysis for exon 8 in the PTEN gene in Hec6 cells

**Additional file 3: Table S2.** Summary of antibodies used in this study

**Additional file 4 Figure S2.** Methylation status of the *cyclin D2* promoter in H6-PTEN and mock cells. *U* Unmethylated; *M* Methylated

**Additional file 5: Fig. S3.** Protein and/or mRNA expression of PTEN and cyclin D2 in Em Ca with morules. **A** HE and IHC staining for PTEN in Em Ca with morules. Note the strong PTEN immunoreactivity in all morular lesions (indicated by arrows). The morule indicated by closed boxes (middle panels) is magnified in the inset (lower panels). Original magnification, x100 and x400 (inset). **B**, **C** Upper: staining by HE, IHC for PTEN, and RNAscope for PTEN (**B**) and cyclin D2 mRNA (**C**) in morular lesions (indicated by arrows) and surrounding carcinoma (Sur Ca) of Em Ca. Note the dot signals for PTEN and cyclin D2 mRNA in both lesions. Original magnification, x200. Lower: number of ISH signals for PTEN (**B**) and Cyclin D2 per cells (**C**) in Em Ca with morules. The data shown are as means ± SDs

**Additional file 6: Table S3.** Relationship between PTEN expression and morular fomation and histological garde of endometrial carcinomas

**Additional file 7: Fig. S4.** HE and IHC staining for the indicated proteins in premorular lesions (indicated by arrows) in Em Ca with morules. Original magnification, x200

**Additional file 8: Fig. S5.** **A** Ishikawa cells were transfected with EBP50 reporter constructs, together with estrogen receptor α (ERα). Relative activity was determined based on arbitrary light units of luciferase activity normalized to pRL-TK activity. The activities of the reporter plus the effector relative to that of the reporter plus empty vector are shown as means ± SDs. **B** Ishikawa cells were transfected with Top reporter constructs, together with EBP50 and β-catenin

**Additional file 9: Fig. S6.** Changes in phenotypic characteristics in EBP50 knockout (KO) cells. **A** Left upper: western blot analysis for the indicated proteins in total lysates from H6-EBP-KO and mock cells. Left lower and right: phase contrast images of H6-EBP-KO cells. Note the changes in cell morphology toward more spread and flattened features in H6-EBP-KO cells. **B** Western blot analysis for the indicated proteins in total lysates from H6-EBP-KO and mock cells

## Data Availability

Data and materials will be shared.

## Abbreviations

PTEN: Phosphatase and tensin homolog deleted on chromosome ten
Em Ca: Endometrial carcinoma
EBP50: Ezrin-radixin-moesin-binding phophoprotein-50
PI3K: Phosphatidylinositol-3 kinase
EMT: Epithelial–mesenchymal transition
CSC: Cancer stem cell
GST: Glutathione *S*-transferase
SA-β-gal: Senescence-associated β-galactosidase
IHC: Immunohistochemistry
Sur Ca: Surrounding carcinoma
ISH: In situ hybridization
ADR: Adriamycin
KO: Knockout
