# Supplementary figures and images for: PTEN overexpression and nuclear β-catenin stabilization promote morular differentiation through induction of epithelial–mesenchymal transition and cancer stem cell-like properties in endometrial carcinoma
Source: Cell Commun Signal. 2022 Nov 21;20:181. doi: 10.1186/s12964-022-00999-w (PMC9677676; doi:10.1186/s12964-022-00999-w)

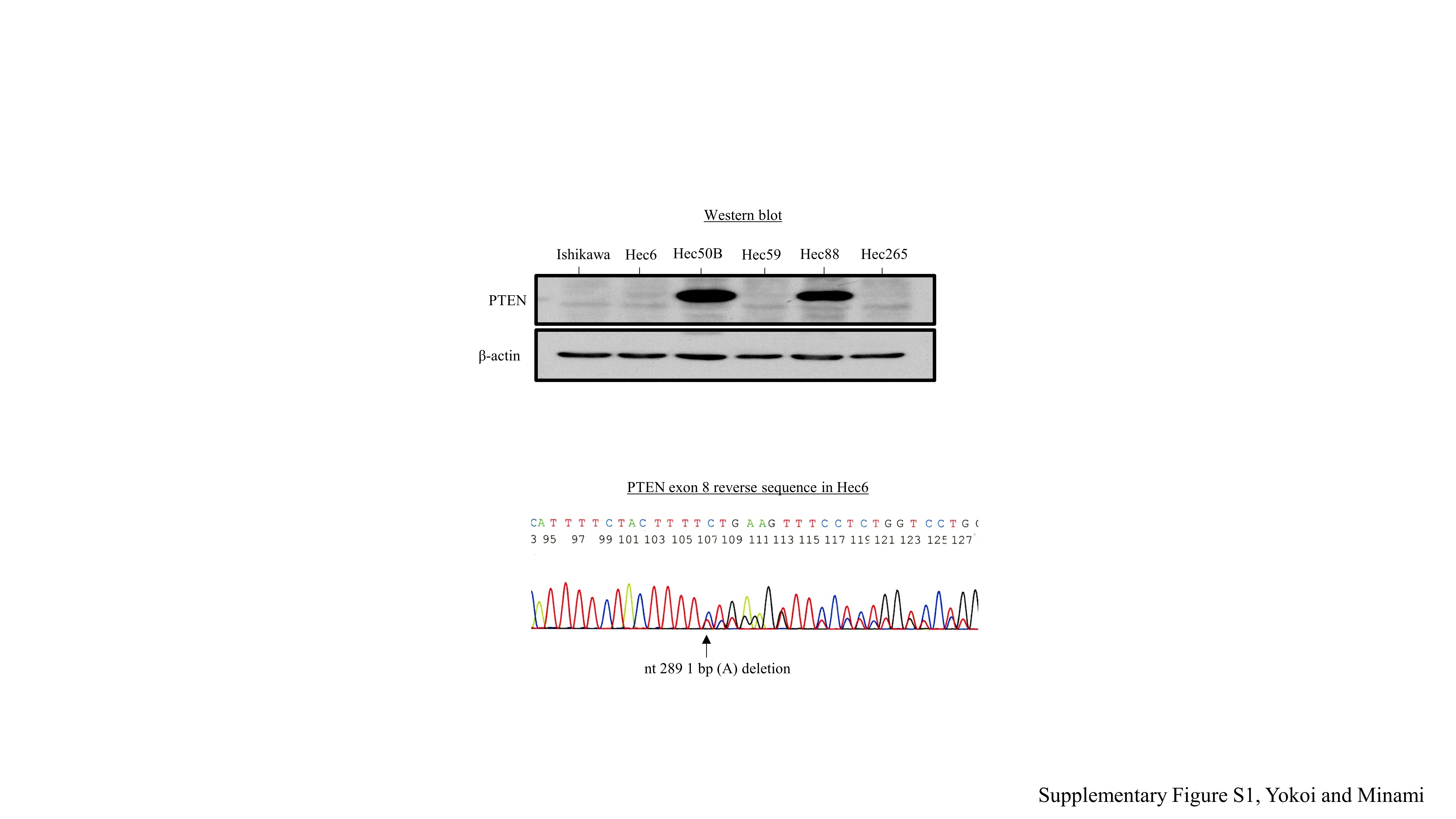

Supplement: Supplementary file 3 — Additional file 2: Fig. S1. PTEN expression and mutation in Hec6 cells. Upper: western blot analysis for PTEN proteins in total lysates from six Em Ca cell lines. Lower: sequencing analysis for exon 8 in the PTEN gene in Hec6 cells [file 12964_2022_999_MOESM3_ESM.tif]

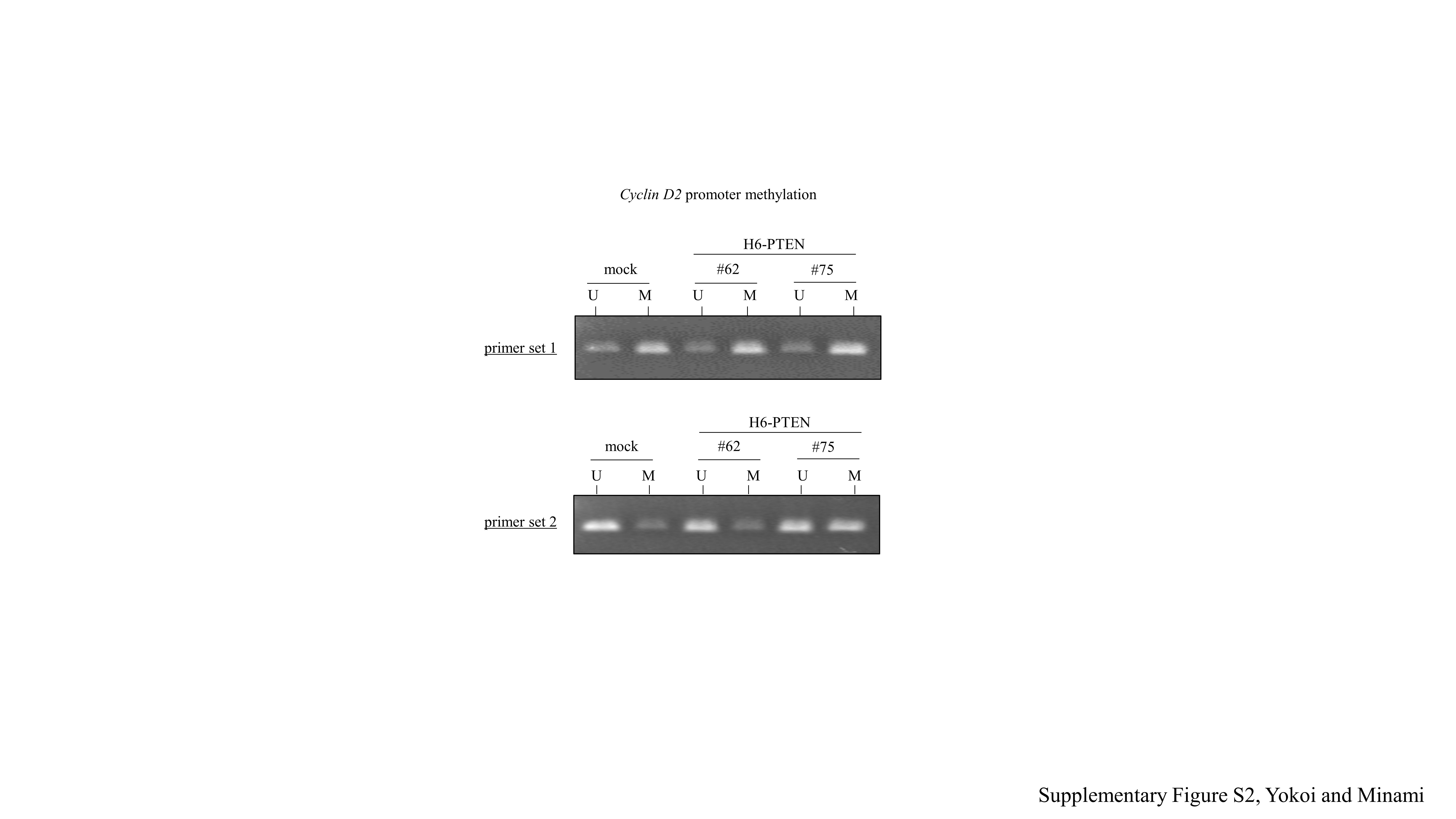

Supplement: Supplementary file 5 — Additional file 4 Figure S2. Methylation status of the cyclin D2 promoter in H6-PTEN and mock cells. U Unmethylated; M Methylated [file 12964_2022_999_MOESM5_ESM.tif]

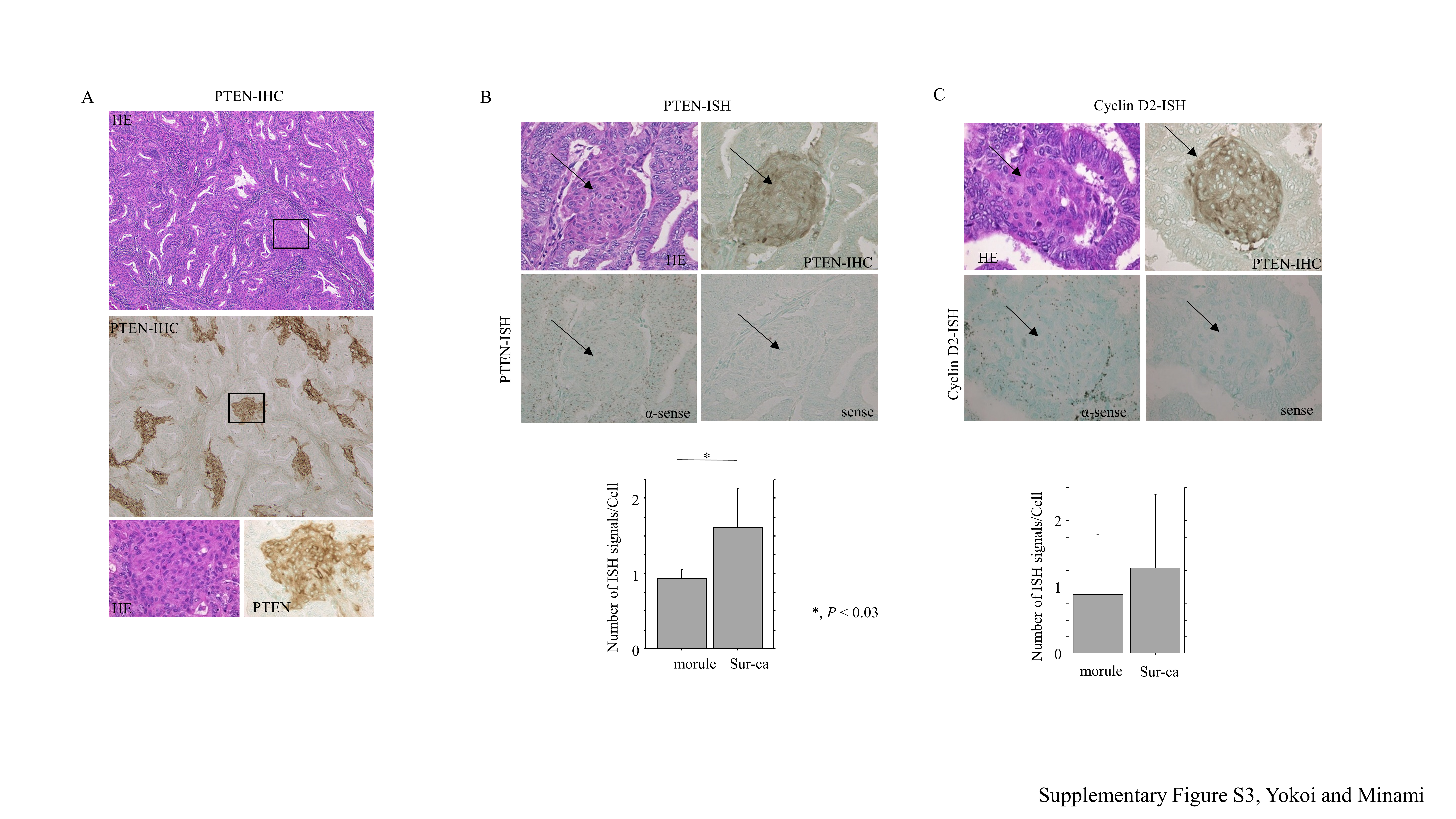

Supplement: Supplementary file 6 — Additional file 5: Fig. S3. Protein and/or mRNA expression of PTEN and cyclin D2 in Em Ca with morules. A HE and IHC staining for PTEN in Em Ca with morules. Note the strong PTEN immunoreactivity in all morular lesions (indicated by arrows). The morule indicated by closed boxes (middle panels) is magnified in the inset (lower panels). Original magnification, x100 and x400 (inset). B, C Upper: staining by HE, IHC for PTEN, and RNAscope for PTEN (B) and cyclin D2 mRNA (C) in morular lesions (indicated by arrows) and surrounding carcinoma (Sur Ca) of Em Ca. Note the dot signals for PTEN and cyclin D2 mRNA in both lesions. Original magnification, x200. Lower: number of ISH signals for PTEN (B) and Cyclin D2 per cells (C) in Em Ca with morules. The data shown are as means ± SDs [file 12964_2022_999_MOESM6_ESM.tif]

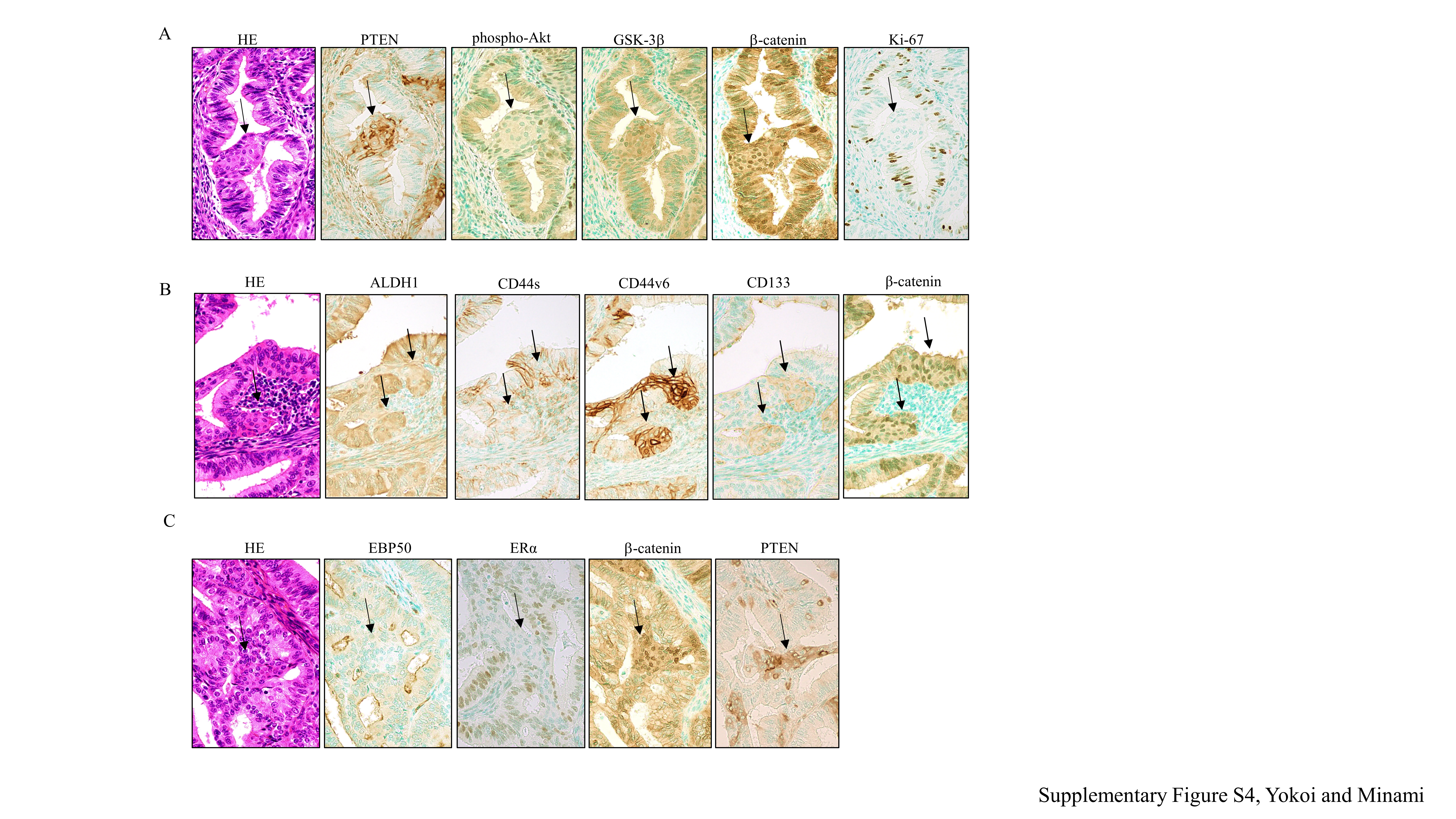

Supplement: Supplementary file 8 — Additional file 7: Fig. S4. HE and IHC staining for the indicated proteins in premorular lesions (indicated by arrows) in Em Ca with morules. Original magnification, x200 [file 12964_2022_999_MOESM8_ESM.tif]

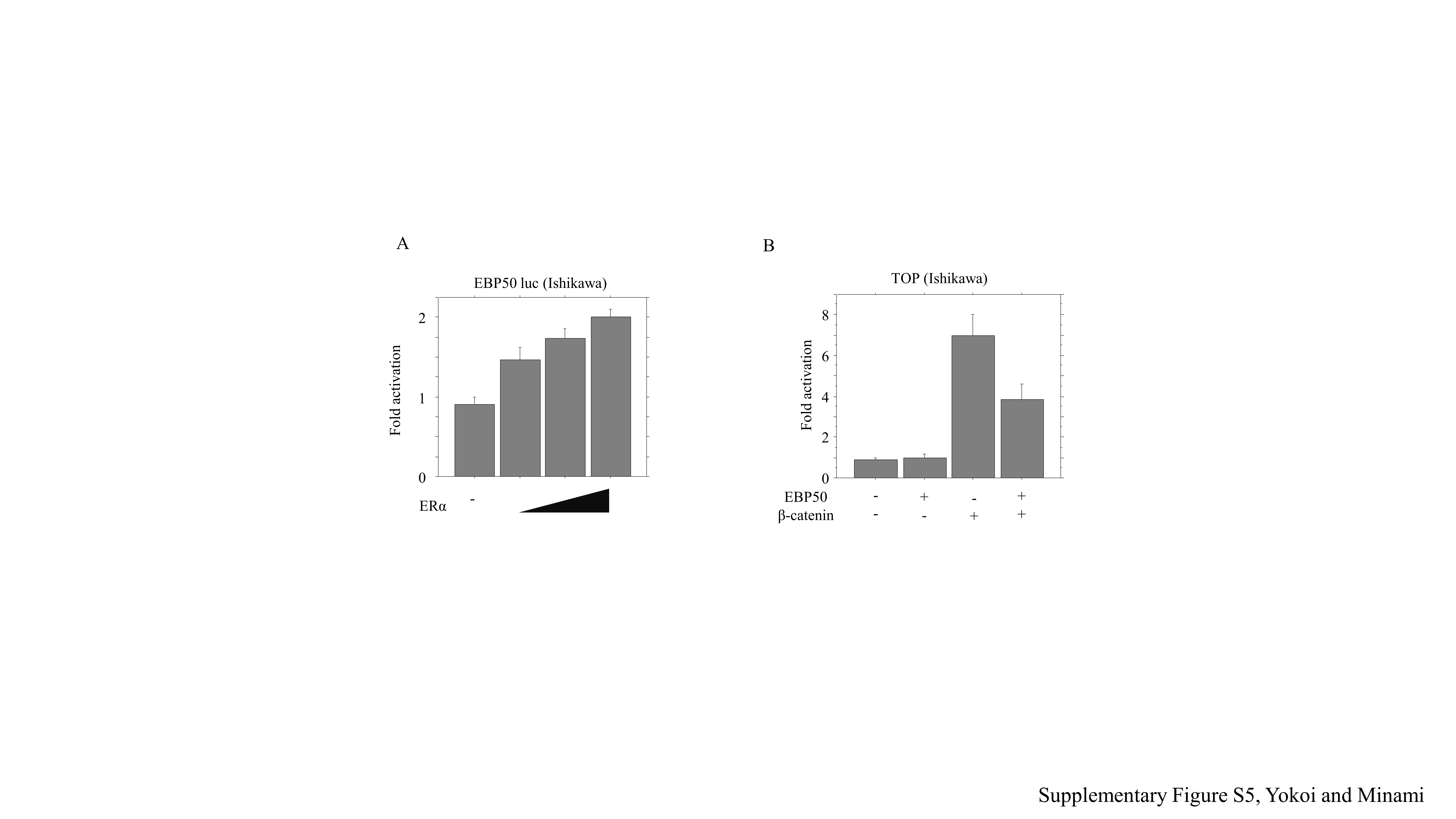

Supplement: Supplementary file 9 — Additional file 8: Fig. S5. A Ishikawa cells were transfected with EBP50 reporter constructs, together with estrogen receptor α (ERα). Relative activity was determined based on arbitrary light units of luciferase activity normalized to pRL-TK activity. The activities of the reporter plus the effector relative to that of the reporter plus empty vector are shown as means ± SDs. B Ishikawa cells were transfected with Top reporter constructs, together with EBP50 and β-catenin [file 12964_2022_999_MOESM9_ESM.tif]

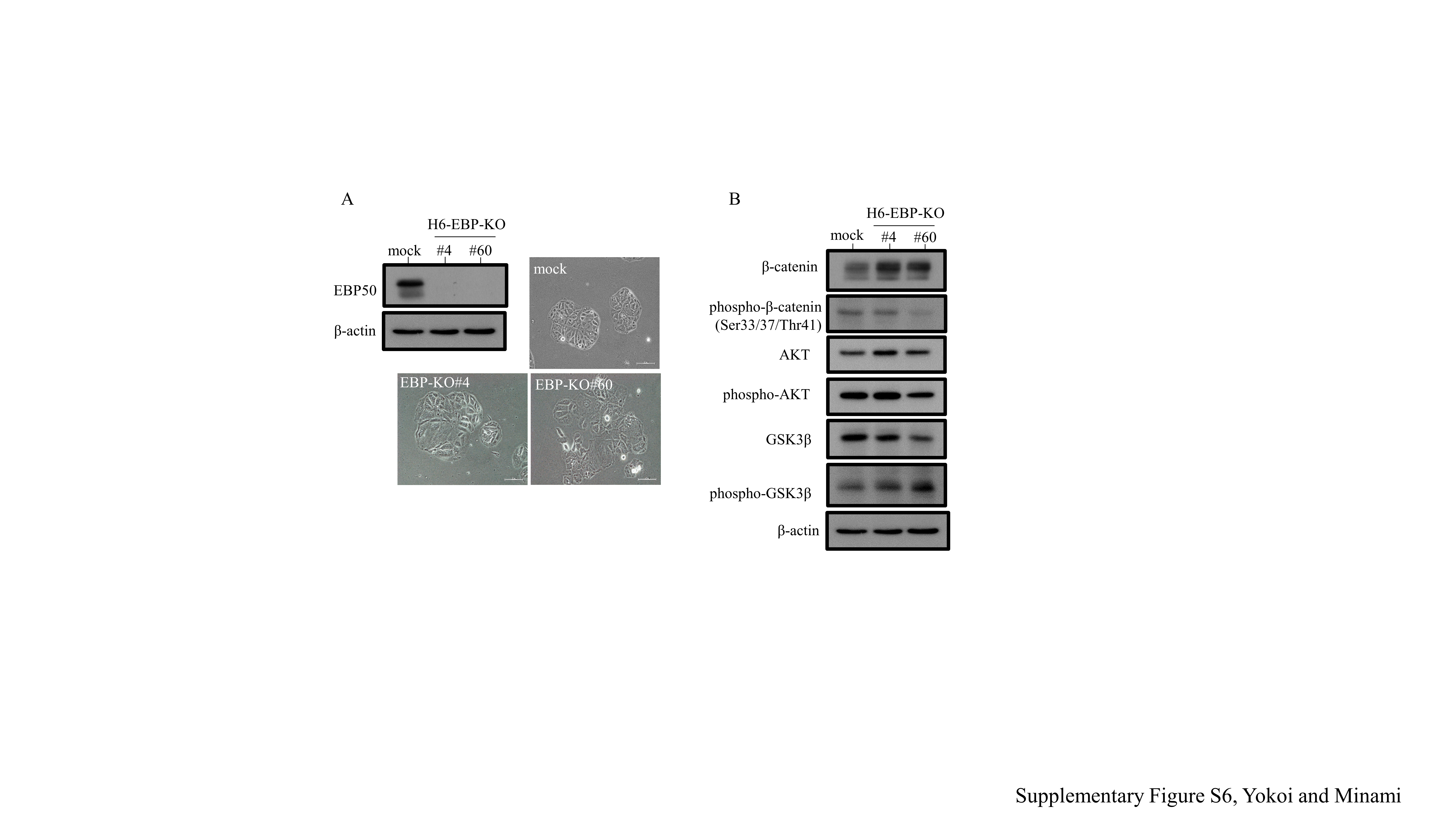

Supplement: Supplementary file 10 — Additional file 9: Fig. S6. Changes in phenotypic characteristics in EBP50 knockout (KO) cells. A Left upper: western blot analysis for the indicated proteins in total lysates from H6-EBP-KO and mock cells. Left lower and right: phase contrast images of H6-EBP-KO cells. Note the changes in cell morphology toward more spread and flattened features in H6-EBP-KO cells. B Western blot analysis for the indicated proteins in total lysates from H6-EBP-KO and mock cells [file 12964_2022_999_MOESM10_ESM.tif]
